# A Wearable-Based Program to Optimise Stress Regulation, Resilience, and Wellbeing in Emergency Care Settings: A Proof-of-Concept Study Protocol

**DOI:** 10.3390/s26010104

**Published:** 2025-12-23

**Authors:** Ilaria Pozzato, Maia Parker, Robyn Tate, Mohit Arora, John Bourke, Matthew Ahmadi, Mark Gillett, Candice McBain, Yvonne Tran, Vaibhav Arora, Jacob Schoffl, Ian D. Cameron, James W. Middleton, Ashley Craig

**Affiliations:** 1 John Walsh Centre for Rehabilitation Research, The Kolling Institute, Northern Sydney Local Health District, St Leonards, NSW 2065, Australia; 2 Faculty of Medicine and Health, The University of Sydney, Sydney, NSW 2006, Australia; 3 Mackenzie Wearables Research Hub, Charles Perkins Centre, The University of Sydney, Sydney, NSW 2006, Australia; 4 Emergency Department, Royal North Shore Hospital, Northern Sydney Local Health District, St Leonards, NSW 2065, Australia; 5 Cities and Planetary Health, School of Architecture, Design and Planning, The University of Sydney, Sydney, NSW 2006, Australia

**Keywords:** psychological stress, stress responses, autonomic nervous system, heart rate variability, sleep, circadian health, wearables, resilience, wellbeing, healthcare, emergency care

## Abstract

Emergency Departments (EDs) are high-pressure environments that place significant psychological and physiological stress on both patients and healthcare staff. Despite increasing awareness of stress-related impacts, proactive stress management interventions have limited uptake in healthcare. This proof-of-concept study will evaluate *WeCare*: a 6-week, wearable-integrated, self-guided program grounded in a “Learn–Track–Act” framework to support stress regulation, resilience, and wellbeing. The study will examine four key aspects of implementing the program: (1) feasibility, (2) acceptability and usability, (3) preliminary clinical effectiveness (self-report and physiological outcomes), and (4) preliminary economic impacts. Using a mixed-methods, multiple-baseline N-of-1 design, the program will be trialled with up to 32 participants across four ED-exposed groups: patients with non-severe or severe injuries, patients with acute medical presentations, and ED staff. The intervention includes digital psychoeducation, continuous biofeedback via a smart ring, personalised guidance, and evidence-based self-regulation strategies. Assessments will include standardised questionnaires combined with continuous physiological monitoring via a smartwatch, and interviews. Quantitative outcomes include heart rate variability, sleep patterns, perceived stress, wellbeing, healthcare use, and time off work. Qualitative interviews will explore user experience, usability, and perceived barriers. The findings will inform the refinement of the intervention and co-design of a larger-scale trial, contributing valuable evidence to support low-cost, wearable-enabled proactive mental healthcare in high-stress healthcare environments.

## 1. Introduction

Emergency care settings are high-pressure environments where both patients and healthcare staff experience significant stress. For patients, this stress may arise from acute injuries, distressing symptoms, or the uncertainty surrounding their condition and need for urgent care [1,2]. For healthcare workers, the high cognitive, physical, and emotional demands of emergency care contribute to sustained work-related stress [3,4,5,6,7]. The interactions between these groups can further influence health outcomes, making stress management a critical factor in emergency care.

While stress is an adaptive response to acute challenges, involving physiological and emotional regulation processes, excessive or prolonged stress is detrimental to health [8]. Heightened stress reactivity is linked with increased morbidity and mortality [8], including higher risks of cardiovascular disease, immune dysregulation, and mental health disorders such as post-traumatic stress disorder (PTSD), depression, and anxiety disorder [8,9,10,11]. The ability to adapt and self-regulate, by efficiently modulating emotional and physiological responses to situations, is considered central to mental health and wellbeing [11,12]. This capability influences how a person copes with stress, maintains daily functioning and life roles, and achieves optimal performance [12,13]. Despite increasing recognition of the significant health impacts of excessive or prolonged stress, particularly on mental health [8,14], there remains a lack of effective, scalable, self-directed interventions for patients and healthcare staff exposed to high-stress environments such as emergency care settings.

Acute injuries [15], particularly those resulting from motor vehicle crashes [16], are major contributors to global mental health burden and disability [17,18,19], often leading to significant psychophysiological distress [20,21]. Studies show that up to 50% of individuals with acute injuries remain highly distressed six weeks after injury, increasing their risk of developing PTSD, depression, and anxiety disorders [9,10,22,23]. Dysregulated stress responses and heightened physiological arousal have also been identified as physiological stress markers in those experiencing elevated distress following traumatic injuries [20,24]. Despite its considerable impact, psycho-physiological distress associated with sustaining an injury often goes unrecognised, delaying timely support during a critical window for recovery. The Emergency Department (ED) is a critical setting for recognising acute stress, raising awareness, and initiating early psychological support, particularly for patients with minor-to-moderate injuries for whom the ED may be the only point of care. While physical stabilisation is essential, the psycho-physiological impact of trauma is equally important and should receive the same priority in ED care. Neglecting emotional consequences leaves many patients unprepared to cope, given their central role in holistic and sustained recovery [11].

ED healthcare staff face unique daily stressors, fatigue, and repeated exposure to trauma, which disrupt autonomic and circadian regulation, increasing their vulnerability to chronic stress and burnout [3,4,5,6,7,25,26]. Research has demonstrated dysregulation of stress response systems in this population, including blunted cortisol responses and autonomic imbalance [27,28]. Despite this, there is a lack of proactive and sustainable stress-management strategies that are specifically tailored to this workforce and integrated into routine clinical workflows. This highlights a critical gap in current approaches to supporting staff wellbeing and organisational resilience, both of which are essential for maintaining high-quality, safe patient care [26,29].

Best practice for stress measurement recommends combining subjective self-reported outcomes and objective physiological indicators [30]. The autonomic and circadian systems are central to stress, sleep, and mental health regulation [31,32,33]. Disruptions in these systems are emerging as early markers of poor mental health, offering new targets for personalised intervention [8,34,35,36,37,38]. The autonomic nervous system, in particular, plays a central role in stress regulation and wellbeing. Heart rate variability (HRV), a key biomarker of stress resilience, has been consistently associated with emotional regulation, vulnerability to stress, stress-related and mental health disorders [20,36,37,38], as well as broader health outcomes [39,40]. HRV is also closely tied to sleep quality [41,42], highlighting the interconnectedness of sleep and stress in maintaining health and wellbeing. Wearable sensor technologies now enable real-time, ecological monitoring of stress, sleep, and autonomic and circadian regulation through biomarkers like HRV and sleep parameters [35,43,44,45]. Evidence shows wearable-based lifestyle interventions can improve stress, sleep, and overall wellbeing [46,47,48,49], including within healthcare settings and among healthcare staff cohorts [50,51,52,53]. These personalised, low-cost tools show promise for high-stress settings, though adoption and sustained use remain challenging, highlighting the need for pilot studies to assess feasibility.

This proof-of-concept study aims to address these gaps by introducing a 6-week wearable-integrated program to support stress regulation and wellbeing: “Wearable-enhanced Care to Assist stress Regulation and Empowerment (WeCare)*”*. Aligned with the first three steps of the Sax Institute’s Translational Research Framework [54], the study will evaluate the program’s (i) feasibility (practicality and acceptability), (ii) preliminary effectiveness (clinical and cost-related impacts), and (iii) replicability. To assess replicability, WeCare will be trialled across four vulnerable ED groups: patients with non-severe and severe traumatic injuries, patients with acute medical symptoms, and ED healthcare workers. The program will be co-designed and adapted for key user groups in Australian ED settings, where mental health needs are frequently under-addressed. The study will generate critical pilot data to refine the intervention and guide future large-scale trials.

### Study Objectives

As this manuscript presents a study protocol, the purpose of this section is to outline the planned study design and objectives. The evaluation of the WeCare program based on experiences in real-world ED settings will focus on four key objectives, to inform a subsequent larger trial designed to include more participants and broader ED settings:

(1) *Feasibility (practicality of study methods):* Assessing the practicality of recruitment and data collection, adherence/engagement to the program, fidelity of program delivery and use, and program safety.
(2) *Acceptability and usability*: Exploring user experience with the program and devices, including perceived benefits, challenges, and suggestions for improvement.
(3) *Preliminary clinical effectiveness:* Evaluating changes in psychological health and wellbeing using self-reported outcomes (e.g., stress, mood, and anxiety) and physiological markers (e.g., HRV, sleep quality, and circadian rhythm).
(4) *Preliminary economic impact:* Estimating the potential cost benefits/savings of WeCare as an early intervention for vulnerable ED populations.

## 2. Materials and Methods

### 2.1. Participants and Setting

Four groups of participants, including patients and ED healthcare workers in emergency care settings, will be recruited from the ED of Royal North Shore Hospital, a Level 1 Trauma Centre, in Sydney, Australia. Between 3 and 8 eligible participants will be recruited per group (at least 3 participants per experiment) for a total of up to 32 participants. The groups are as follows:

- *Sample* 1: Patients presenting to the ED with mild-to-moderate injuries (Injury Severity Score, ISS < 15).
- *Sample* 2: Patients presenting to the ED with severe injuries (ISS ≥ 15).
- *Sample* 3: Patients presenting to the ED with acute non-traumatic, minor-to-moderate medical complaints (Australasian Triage Scale, ATS category 3–5 = less urgent).
- *Sample* 4: Emergency healthcare workers exposed to workplace stress.

### 2.2. Design Overview

**Figure 1:**
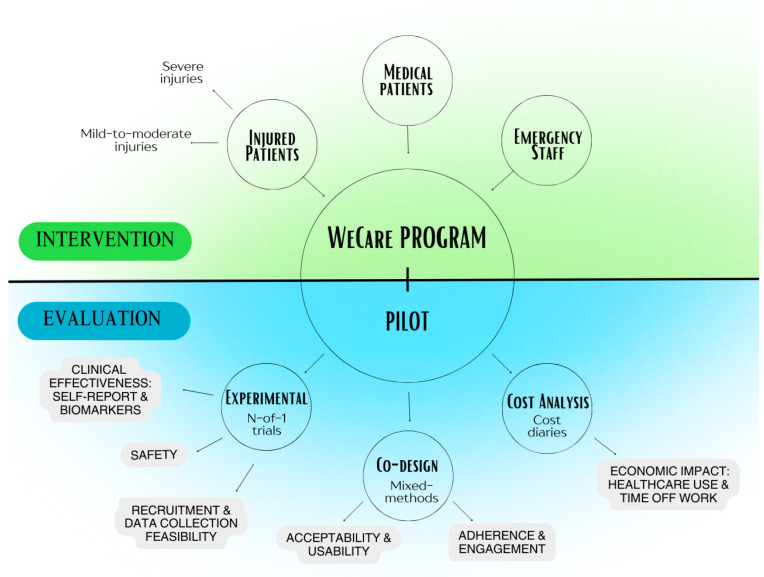
Flow map of the WeCare program pilot evaluation in emergency care settings.

The study includes three interrelated components: (1) an Experimental component, with nested (2) Co-design and (3) Cost analysis components. Each component will address distinct study objectives and evaluation aspects, [54] using different methodologies, to pilot the 6-week WeCare program in a controlled setting, as illustrated in Figure 1.

#### 2.2.1. Experimental Component

**Figure 2:**
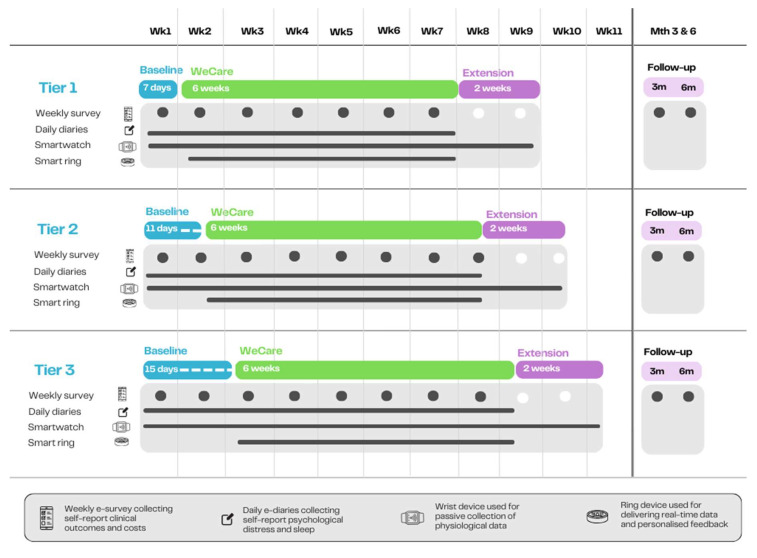
Experimental design and timeline of a single-case multiple-baseline experiment across participants with three tiers. Participants begin a 1–3-week baseline (usual care) phase, followed by a 6-week WeCare intervention. A staggered multiple-baseline design offsets intervention start: Tier I ~ Day 7 (1st week), Tier II ~ Day 11 (2nd week), Tier III ~ Day 15 (3rd week), providing within-person controls until the intervention begins.

The Experimental component aims to evaluate the *preliminary clinical effectiveness* of the program, the *feasibility of recruitment and data collection methods*, and the *safety of the intervention* (adverse events) across the target participant groups (Figure 1). A series of N-of-1 trials will be conducted, nested in a randomised, concurrent multiple-baseline experiment design across participants with at least three tiers (Figure 2). The study will incorporate both direct inter-subject and systematic replications, with a minimum of three participants (tiers) per replication experiment. The protocol for these trials was guided by the Risk of Bias in N-of-1 Trials (RoBiNT) Scale [55,56]. N-of-1 trials are individual randomised controlled trials in which each participant serves as their own control. This design is well suited for application in heterogeneous conditions [57] like stress-related conditions due to its sensitivity to individual differences. When aggregated, N-of-1 trials can yield population-level treatment estimates comparable to traditional randomised controlled trials [58], but with greater efficiency and cost-effectiveness, as they require fewer participants. Quantitative methods will be used for this experimental component of the study, including patient self-report and physiological data from smart wearables.

**Experimental phases:**

Table 1 and Figure 2 provide a schematic overview of the experimental study design, phases, and timeline. Each single-case experiment, including at least three tiers, begins with a **(i) Baseline phase (usual care)** of 1–3 weeks, with duration randomised for each participant, followed by a **(ii) Intervention phase (WeCare program)** of 6 weeks. A staggered, multiple-baseline design is used: all participants start the baseline at the same time, but the intervention begins at different times (Tiers I–III)—approximately 7 days (end of the 1st week) for Tier I, 11 days (midpoint of the 2nd week) for Tier II, and 15 days (start of the 3 week) for Tier III. This ensures robust within-person controls and reducing external bias. The minimum 7-day baseline allows for the collection of at least five observations, ensuring maximum rigour [59], while accommodating day-to-day variability (e.g., “bad day”) and logistical constraints (e.g., participant availability for an in-person visit). During the Baseline phase, participants will be provided with a *smartwatch* device (Vivoactive 5, Garmin Ltd., Schaffhausen, Switzerland, https://www.garmin.com/en-AU/legal/compliance/, https://www.garmin.com/en-AU/p/1057989/; accessed on 15 October 2025), worn on the wrist for continuous physiological monitoring through the whole study. The device’s display will remain blank, and participants will not have access to any feedback or vital sign data. They will be instructed to continue their daily routines and usual care practices without changes to medication, lifestyle, or activities. To support retention and maintain engagement during the baseline phase, participants will receive one to two brief messages per week acknowledging their data collection and encouraging continued participation. Regular contact of this kind has been shown to improve adherence and reduce attrition in single-case and digital health intervention studies [60].

At the start of the Intervention phase, participants will receive a *smart ring* device (OuraRing4, Oura Health, Oulu, Finland, https://ouraring.com/terms-of-use, https://ouraring.com; accessed on 15 October 2025), which will serve as their companion device for the *WeCare* program. Unlike the smartwatch, the smart ring allows participants to view their physiological data in real time through an associated app and provides personalised guidance to support behavioural and lifestyle modifications. Each of the above phases will begin with an in-person session that includes participant education, device setup, and usage instructions. Alternatively, this session may be delivered via telehealth or through a pre-recorded video, with the device mailed or couriered to participants. Following the intervention, an optional **(iii) Extension phase** of 2 weeks will be offered, allowing participants to experience the program using the smartwatch as an alternative wearable. This phase will be an integral part of the “acceptability” evaluation conducted during the co-design component and is not intended to test effectiveness. Follow-up assessments will be conducted at 3 and 6 months post-intervention.

**Replication:**

Replication is essential in single-case experimental designs to strengthen internal and external validity by confirming causality and generalisability of the results. It helps reduce uncertainty, especially when different visual criteria or statistical methods yield conflicting conclusions [61]. This study uses both direct and systematic replication. **Direct replication** tests the reliability of effects within a similar population (i.e., inter-subject replication) [62]. In our study, this involves conducting another multiple-baseline experiment with at least three tiers within the same participant group. This is particularly important for ED patients with mild-to-moderate injuries (*Sample* 1, ISS < 15), who serve as the reference population, ensuring consistency across individuals with comparable presentations. **Systematic replication**, by contrast, typically involves modifying aspects of the original study, such as the setting, behaviour, participants, and components of the intervention [63]. In this study, replicability will be examined by applying the intervention to three additional groups within the emergency care setting:

- *Sample* 2: More severely injured participants (ISS ≥ 15).
- *Sample* 3: Non-injured patients with acute stress from medical events.
- *Sample* 4: Health workers from the ED exposed to workplace stress.

By varying participant characteristics and stressors, systematic replication helps determine whether the intervention’s effects extend beyond the initial sample, enhancing its relevance across diverse real-world emergency care contexts. This dual approach strengthens both the reliability and broader applicability of the intervention.

#### 2.2.2. Co-Design Component

The nested, Co-design component, whereby participant perspectives have the capacity to alter and shape the larger-scale trial, aims to evaluate the feasibility of the intervention. *Participants’ adherence, preliminary engagement,* and *satisfaction* with the WeCare program and wearables used will be examined, including the user *acceptability* and *usability* based on individual experience (Figure 1). Mixed methods will be used for this component, including quantitative approaches that provide objective feasibility and acceptability data, complemented by qualitative interviews/focus groups. Participants from each sample group will be invited to provide rich, detailed insights into their experiences (see Section 2.6.3 for details). The findings will inform the co-design of a user-centred solution tailored to the needs of these populations and guide the development of a larger-scale trial for future implementation and scalability.

#### 2.2.3. Cost Analysis Component

The Cost analysis component aims to evaluate the *preliminary cost benefits* of implementing the WeCare program by quantitatively assessing changes in healthcare use and time off work (Figure 1). In this preliminary study, this data will be obtained through self-report measures, including participant diaries on frequency of healthcare visits, medication use, and number of sick leave days taken. While self-reporting provides an accessible and low-burden approach for early-stage evaluation, it will also inform the feasibility of incorporating objective cost and utilisation data (e.g., administrative records) in future larger-scale trials. It will examine whether the initial investment results in cost savings, helping to determine if the program is a viable, low-cost, and sustainable solution for improving mental wellbeing and resilience in emergency care settings.

### 2.3. Eligibility Criteria

Each sample of participants will be participating in all three components of the trial. The inclusion and exclusion criteria for each of the four samples are detailed in Table 2.

### 2.4. Recruitment, Enrolment, and Randomisation

Patients (*Samples* 1–3) presenting to the RNSH ED will be consecutively screened for study eligibility by ED personnel based on information available on the electronic medical record system. Individuals who are deemed eligible will be informed about the study and invited to participate either during their ED visit or by follow-up call or email. The ED personnel will be provided with an ethically acceptable script to use when approaching participants. If individuals express interest in the study, the ED personnel will ask permission to share their contact information with the research team. A member of the research team will contact them for further screening, and potential participants will also have the opportunity to ask questions about the study. ED healthcare workers (*Sample 4*) will be recruited through flyers displayed on ED noticeboards and wards, email invitations, or direct clinical contact with support from the ED Wellness Group at RNSH. All interested ED healthcare worker participants will be screened by the research team against the eligibility criteria before enrolling them in the study.

All participants who meet the study inclusion criteria will be provided with comprehensive study information, both verbally and through a written study advertisement and participant information sheet. Potential participants can take their time to think and decide, and if they agree to enrol in the study, they will be asked to sign a written informed consent form via a secure online REDCap platform, hosted by The University of Sydney. Potential participants will also be asked their preferred day and time to attend the introductory study session at the start of the baseline phase, where they will receive their smart watch and orientation to the study.

Once a cohort of at least 3 eligible participants is recruited, they will be enrolled concurrently, assigned a unique study number, and booked in for the introductory session. Participants will be randomly allocated to a baseline phase duration of 1, 2, or 3 weeks (i.e., 7, 11, or 15 days, respectively) depending on their tier, in accordance with the study’s single-case design standards. An independent researcher, not involved in participant recruitment, will generate a randomisation sequence using standard computerised methods to minimise bias. Randomisation will take place after the pre-baseline data is collected (Week 0) to reduce the risk of allocation bias. Investigator blinding is not feasible due to study procedures, and this is not a randomised clinical trial.

All participants who choose to enrol in the study will be automatically included in all three components of this proof-of-concept study. Upon completion of the *WeCare* program as the experimental component, research investigators will invite participants to take part in in-depth interviews. Additionally, cost data will be collected weekly throughout the duration of the study.

### 2.5. Intervention: WeCare Program

WeCare is an innovative, evidence-based, wearable-integrated wellbeing initiative designed for individuals exposed to significant stress. It delivers a 6-week, self-directed and self-reflective intervention program aimed at improving stress regulation, enhancing resilience, promoting sustainable recovery, and optimising performance and wellbeing. The program integrates biometric wearable monitoring with psychoeducation and personalised behavioural guidance to mitigate the risk of disability associated with acute (e.g., injury, trauma, or illness), chronic, or occupational stress (e.g., ED workers).

Co-designed by a multidisciplinary team of experts in mental health, injury recovery, and rehabilitation, together with individuals with lived experience, WeCare empowers participants to take charge of their health by combining real-time physiological data from wearables and behavioural science through personalised, data-informed feedback, and expert guidance.

#### 2.5.1. Wearable Integration and Personalised Data-Driven Feedback

The program employs an advance *smart ring* device (OuraRing4, Oura Health, Oulu, Finland, https://ouraring.com/terms-of-use, https://ouraring.com; accessed on 15 October 2025) to provide continuous physiological feedback to participants. Using infrared LEDs, temperature sensors, an accelerometer, and a gyroscope, the ring collects real-time biometric data, including key indicators such as HRV, respiratory rate, sleep metrics, and body temperature.

The associated app applies proprietary algorithms, which incorporate elements of machine learning/artificial intelligence to analyse the data. The app summarises daily and weekly trends, generating health summary scores related to stress, recovery, and activity. Participants will receive daily personalised, data-driven insights based on their individual physiological patterns. These insights, paired with guided reflections and practical suggestions, will support participants’ understanding of their stress and recovery cycles, as well as how their habits influence their health trajectory, encouraging self-awareness and adaptive behaviour.

#### 2.5.2. Program Framework: The “Learn–Track–Act” Paradigm

**Figure 3:**
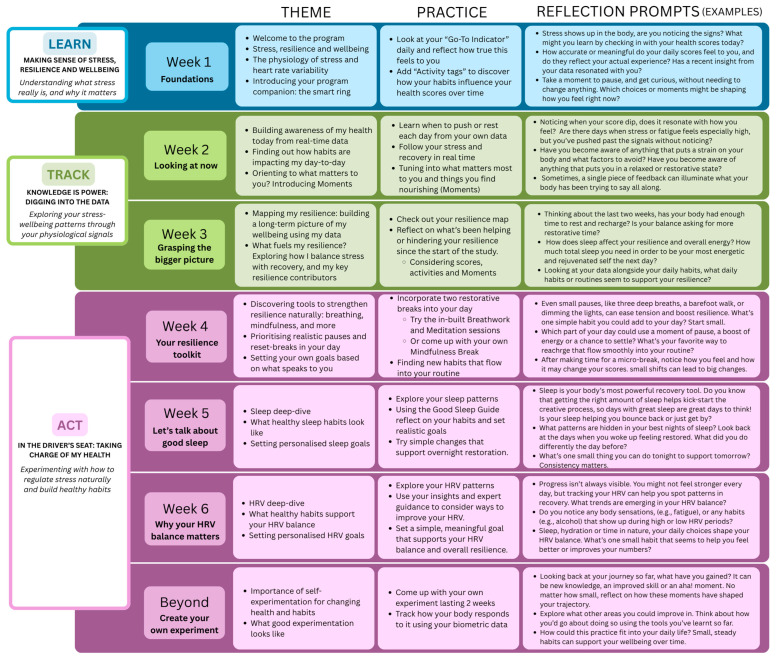
WeCare program: weekly themes, suggested activities, and reflection prompts.

The WeCare program uses a structured “Learn–Track–Act” paradigm (Figure 3), guiding participants through a stepped, personalised process of self-awareness and behaviour change. This framework is embedded across the 6-week intervention and involves the following aspects:

- *Learn:* Participants engage with digital psychoeducational content on stress, wellbeing, and self-regulation strategies via factsheets, videos, and real-life stories.
- *Track:* Participants monitor their daily physiological data, observe trends over time, and see how their activities influence key biometrics and how they feel, using the wearable-based app.
- *Act:* Participants receive personalised feedback via the app and expert guidance based on their data and engage in evidence-based self-regulation practices (e.g., lifestyle adjustments, paced breathing, and mindfulness) to support stress management and recovery.

#### 2.5.3. Program Structure

Participants will be guided through the following steps (Figure 3):

(1) *Making sense of stress and wellbeing* (Week 1)

Participants attend a 60–90 min psychoeducation session, delivered in person at the RNSH laboratory, or via telehealth as a pre-recorded video. This session introduces the multidimensional nature of stress and wellbeing, including its physiological manifestations. Participants are introduced to the smart ring, instructed on installation of the app, and begin continuous data and daily activity tracking. The goal of this session is to build foundational awareness and context-specific understanding of stress and wellbeing.

(2) *Knowledge is power: digging into the data* (Weeks 2–3)

Participants explore their personal biometric data to improve body awareness and recognise stress and recovery patterns. This phase encourages reflection through guided prompts and educational content, helping participants identify how stress manifests in their physiology and daily routine. The aim is to foster insight and engagement with one’s own health data as a tool for learning and growth, supporting meaningful changes. A 2-week period is required by the smart ring system to calibrate personalised recommendations for each participant.

(3) *In the driver’s seat: taking charge of my health* (Weeks 4–6, and beyond)

This final phase encourages behavioural change through experiential learning. Participants learn about healthy habits and are encouraged to self-reflect and experiment with a range of self-regulation strategies to manage stress and enhance wellbeing, such as the app’s in-built breathwork and mindfulness modules, and integrate these practices into their daily routines. Following Week 6, participants will have the option to continue the intervention for an additional two weeks using a smartwatch instead of the smart ring. Guidance will also be provided on how to design and run a personalised self-experiment beyond the core 6-week program, encouraging sustained engagement and long-term habit formation.

#### 2.5.4. Weekly Insights and Daily Practice Guidance

In addition to daily personalised data-driven feedback via the wearable app, participants will receive structured guidance through a companion digital health platform (accessible via mobile app or desktop) over the 6-week program. This guidance is delivered through a combination of weekly and daily notifications aligned with the program’s core themes to support wellbeing, self-reflection, and sustained behaviour change.

- *Weekly thematic insights*: Each week, participants are provided with evidence-based content aligned with the program’s focus. This includes short readings, video resources, e-learning links, and lived-experience stories. Weekly modules also offer practical guidance to support daily wellbeing practices and activities such as journaling, breathwork, mindfulness, and lifestyle modifications.
- *Daily reflection prompts:* To encourage engagement and deepen learning, participants receive structured prompts that support reflection on personal experiences, habits, and progress. These prompts aim to foster self-awareness and active participation in the program and behaviour change.

Details of weekly themes, suggested activities, and reflection prompts are presented in Figure 3.

#### 2.5.5. Human and AI-Based Support

Participants will receive a brief (10–15 min) weekly check-in with a qualified member of the research team, delivered via phone or telehealth. These check-ins are designed to monitor progress, address questions, and provide personalised support. Participants may choose to share their biometric data from the smart ring device in advance to help guide these conversations and tailor feedback.

In addition to human support, the wearable app features a built-in, AI-powered smart advisor that offers 24/7 real-time guidance. This advisor provides personalised suggestions based on the participant’s ongoing biometric data and app interactions.

This blended model, combining human with AI-driven support, has been shown to enhance engagement and improve outcomes compared to digital-only interventions [49,64].

### 2.6. Outcome Measures and Data Collection

Outcomes include measures that assess the four study objectives: *Feasibility (practicality of methods), Clinical effectiveness, User acceptability*, and *Cost benefits*.

#### 2.6.1. Feasibility Outcomes

Feasibility outcomes include assessment of recruitment and data collection processes, intervention safety, participant adherence and engagement, and fidelity of program delivery and use. *Recruitment feasibility* and the *suitability of data collection methods* will be evaluated through screening logs, enrolment and retention rates, reasons for non-participation or dropout, and the proportion of missing data. A retention rate of ≥80% will be considered feasible, following traffic light criteria (green: ≥80%, amber: ≥60%, red: ≤40%) [65]. The *safety* of the intervention will be monitored via weekly self-reported adverse events. Participants will be closely monitored for any physical, psychological, or device-related issues, which will be documented and reported according to institutional protocols, contributing to the limited evidence base on the safety of digital and wearable-based interventions. *Adherence and engagement* will be assessed through smart ring wear time and usage analytics from the digital health platform, respectively, as well as users’ feedback, which will also inform evaluation of program fidelity and adherence to intervention components.

#### 2.6.2. Clinical Effectiveness

Clinical effectiveness outcomes will be assessed as part of the experimental component and collected at varying time points, including (a) Pre-baseline, (b) Primary outcome, and (c) Generalisation measures. These include a combination of clinical self-report and psychophysiological outcomes. *Clinical self-report outcomes* will be collected online via a secure database (REDCap) or, where necessary, administered via telephone interview or postal return, while *Psychophysiological outcomes* will be collected continuously and passively via a smartwatch with data securely transmitted and automatically integrated into a digital health platform accessible to the research team. All self-report and physiological data will be managed in compliance with relevant privacy regulations (e.g., GDPR) and securely stored on password-protected platforms, with de-identification applied for analysis and reporting to ensure participant confidentiality. The full list of outcome measures is presented in Table 3.

**(a)**  **Pre-baseline measures**

Collected once at enrolment (Week 0), prior to entering the baseline phase. These include socio-demographic characteristics, medical history, and psychosocial profile (e.g., Psychosocial Index [66], Pain Catastrophising Scale [67], and General Self-Efficacy Scale) [68].

**(b)**  **Primary outcome measures**

Primary outcomes are the target behaviours directly addressed by the intervention. These are assessed daily, throughout the baseline and intervention phases (including at enrolment, Week 0), post-intervention (within 2 weeks of the final session), and at 3- and 6-month follow-ups. They include the following clinical self-report (daily diary) and psychophysiological outcomes of stress regulation, selected for their sensitivity to change and appropriateness for single-case designs.

***Clinical self-report outcomes:***
  1. *Psychological distress*: Daily ratings of 24 h average distress measured with the 1-item Subjective Units of Distress Scale (SUDS), ranging from 0 (no distress) to 100 (extreme distress) [69], plus ecological momentary assessments every 2 h for 4 days only (2 during baseline and 2 during intervention).
  2. *Sleep quality*: Measured each morning using a single-item scale from 0 (worst) to 10 (best), aligned with self-reported wake times to minimise recall bias.
***Psychophysiological outcomes*:** Continuously recorded via a smartwatch using photoplethysmography and an accelerometer. Adherence and data completeness will be monitored via app analytics and automated tracking tools.
  1. *Heart rate variability (HRV) metrics*: Reflects autonomic nervous system (ANS) activity. Higher parasympathetic (vagal) tone indicates improved regulation, whereas low HRV is associated with mental health risk [37,38,39]. HRV Root Mean Square of Successive Differences (HRV-rMSSD) and other time-, frequency-, and nonlinear-domain and composite metrics will be extracted from beat-to-beat intervals, especially during overnight monitoring.
  2. *Sleep parameters*: Includes sleep duration, bedtime, sleep onset latency, nocturnal heart rate, wake-up time, and sleep-related temperature deviation (if available).

**(c)**  **Generalisation measures**

Generalisation measures aim to assess broader impacts of the intervention on participants’ lives.

**‘Proximal’ generalisation measures**

Assessed *weekly* throughout the baseline and intervention phases (including at enrolment, Week 0), post-intervention (within 2 weeks of the final session), and at 3- and 6-month follow-ups (~15 min per completion). They include the following clinical self-report and physiological measures.

***Clinical self-report outcomes*:**
  - *Mental health:*
    ○ Perceived Stress Scale (PSS) [70].
    ○ Patient Health Questionnaire-9 (PHQ-9) [71].
    ○ PTSD Checklist for DSM-5 (PCL-5) [72].
    ○ Generalised Anxiety Disorder Scale (GAD-7) [73].
  - *Sleep quality*: Pittsburgh Sleep Quality Index (PSQI) [74].
  - *Consumer perception of change*: A 15-point Global Impression of Change Scale (−7 to +7).
  - *Overall health status*: EQ-5D visual analogue scale (0–100).
***Psychophysiological outcomes*:** Continuously monitored via a smartwatch, including heart rate, respiratory rate, blood oxygen saturation (SpO_2_), activity metrics (e.g., steps, movement, calories, and circadian rhythm), and daily health summary metrics related to stress and daytime and night-time recovery.

**‘Distal’ generalisation measures**

Assessed at pre-intervention, post-intervention (within 2 weeks of final session), and at 3- and 6-month follow-ups. These aim to assess broader impacts of the intervention on participants’ lives (~15 min per completion) and include measures of general wellbeing, pain interference, social participation, and perceived benefits, etc.

#### 2.6.3. Acceptability and Usability

Acceptability and usability based on user experience of the WeCare program will be evaluated as part of the co-design component that will commence following the conclusion of the WeCare program. The focus is on perceived benefits, unintended effects, challenges encountered, and potential solutions, as reported by each participant group in the study. To guide this evaluation, the “Theoretical Framework of Acceptability (TFA)” [75,76] will be applied. Using the TFA at this stage enables a structured assessment of both anticipated and experienced acceptability for both the recipients and deliverers of the intervention. This will support evidence-informed decisions about refining the program’s form, content, and delivery mode.

Given that this is the first piloting of the WeCare program, the TFA will play a crucial role in identifying whether early expectations align with actual user and provider experiences. Where applicable, adaptations to intervention components (e.g., due to lower-than-expected recruitment or higher attrition) will be explored based on insights from this phase. Acceptability will be operationalised across the seven TFA constructs using a sequential mixed-methods approach, which allows key patterns identified in the quantitative data to be further explored and contextualised through qualitative insights.

- *Quantitative methods*: Questionnaires or visual analogue rating scales aligned with TFA constructs will assess anticipated acceptability among potential deliverers and recipients.
- *Qualitative methods:* Semi-structured interviews or focus groups, guided by the seven TFA constructs, will explore participants’ and stakeholders’ lived experiences of the intervention. These qualitative methods will offer in-depth insights into barriers, facilitators, and contextual factors influencing engagement, adherence, and perceived value, factors often not captured through quantitative means alone.

Participants from the quantitative phase will be invited. In the instance of too many (>10) participants expressing interest in the qualitative phase, researchers will select a sample of 6–10 through discussion, ensuring that at least one participant from each group is included, in line with the recommended sample size by Braun and Clarke [77]. To minimise bias, interviews will, where possible, be conducted by independent researchers who were not involved in delivering the intervention, so that interviewees are unfamiliar with the interviewers. All interviews will be audio-recorded and transcribed verbatim. The transcribed interviews will be analysed using the six-phase approach outlined by Braun and Clarke [77,78], which combines both deductive and inductive processes. This means that while data collection and analysis will be guided by the TFA framework, the process will remain open to novel and emerging concepts arising from the data. Findings from this qualitative phase will directly inform refinement of the *WeCare* program and its future implementation strategies.

#### 2.6.4. Cost Benefits

A nested Cost analysis of the WeCare program will be conducted using weekly self-report diaries collected throughout the study. Data will include *healthcare utilisation* (e.g., medical appointments, hospital visits, treatments, medications, and diagnostics) and *work-related costs* (e.g., time off work due to stress, injury, or illness). The analysis will assess whether the wearable-based intervention reduces healthcare use and work absenteeism. This will inform the economic benefits of implementing the WeCare program as a scalable solution.

### 2.7. Sample Size

Up to 32 participants will be recruited for this pilot study, up to 8 participants in each study group. Power calculation is not relevant as this is an N-of-1 pilot study using a design that has not been utilised previously.

### 2.8. Data Analysis and Reporting

For the primary measures, graphical representation and calculation of a mean and trend line will be used to analyse changes over time and between periods. Trend analyses will also be conducted to account for unstable baselines (e.g., increasing or decreasing severity over time), providing a more nuanced understanding of change. Level- and slope-change analyses will be used to further analyse change between periods. Secondary analyses will be performed on secondary outcomes. No interim analyses will be performed in this study. The level of significance to be used will be set at *p* < 0.05 unless otherwise required. The quality of the dataset will be inspected regularly by outcome assessors for missing data. Additionally, missing or spurious data will also be inspected by one of the senior researchers. Moreover, linear mixed-effects modelling [79], a multiple regression technique that is flexible and suitable for single-case design studies, will be used to provide standardised effect sizes and measures of statistical significance. Analyses will also include evaluation of overlap between phases; for example, Tau-U will be applied as a non-parametric effect size method appropriate for single-case experimental designs.

A feasibility analysis will be also conducted using prespecified progression criteria to help guide the interpretation of pilot trial findings to decide whether, and how, a definitive RCT is feasible and should be conducted. Once all the text information from interviews is collated, qualitative data analysis will be conducted, which involves thematic analysis and directed content analysis to determine the most important themes that emerged from the participant discussions regarding the acceptability of the intervention. A summary of the results will be sent to the participants for checking, verification, and confirmation of the researcher’s interpretation of key issues and thematic understanding. The software package NVIVO will be used to analyse the qualitative data.

To ensure clarity and transparency in reporting, study findings reporting will adhere to the Single-Case Reporting guideline In BEhavioural interventions [80] and Consort Extension for reporting N-of-1 trial guidelines [81], while the qualitative component will be reported following the Consolidated Criteria for Reporting Qualitative Research (COREQ), a 32-item checklist for interviews and focus groups [82].

### 2.9. Study Approvals

The conduct of this study was approved by the Northern Sydney Local Health District HREC (REGIS ID: 2025/ETH00118). The protocol for this study has been prospectively registered with the Australian New Zealand Clinical Trails Registry (ACTRN12625001354471, https://anzctr.org.au/ACTRN12625001354471.aspx, accessed on 19 December 2025).

## 3. Discussion

Severe stress and burnout in ED settings is prevalent and a growing concern, with significant implications for patient and staff wellbeing, the healthcare system, and public health. However, there remains a lack of evidence-based preventative proactive stress management strategies that are effective and accessible within healthcare settings. The WeCare proof-of-concept study aims to evaluate the feasibility, acceptability, and preliminary effectiveness of a wearable-integrated, self-guided program designed to improve mental and physiological wellbeing and stress regulation in high-stress clinical environments, such as ED settings. The program combines digital education, evidence-based stress reduction techniques, and real-time biofeedback via wearable sensor technologies to support self-regulation in day-to-day routine.

WeCare represents a forward-thinking self-managed model for early mental and physical health intervention that can be easily embedded in healthcare and, to our knowledge, has not previously been attempted. It leverages low-cost technology to address long-standing barriers such as mental health clinician shortages, long waitlists, stigma, and limited access to preventive care. By integrating evidence-based tools into a user-friendly, self-managed platform, it offers a scalable and promising solution for tackling distress and building resilience among vulnerable groups in high-stress healthcare environments.

The study will be conducted in a metropolitan hospital in Sydney, focusing on presenting patients with acute conditions (i.e., acute injury or medical complain) and frontline staff who are regularly exposed to high stress in this environment. Quantitative feasibility outcomes, including recruitment, retention, adherence, and data completeness, will help determine the program’s practical implementation in a real-world healthcare setting. Exploratory outcomes will assess changes in psychophysiological sources of distress, emotional exhaustion, sleep, mood, and perceived health, as well as healthcare use and work functioning. A qualitative component will capture participants’ experiences with the program, identifying enablers and barriers to engagement, which will guide future refinement and delivery. Importantly, any adverse events will be closely monitored and reported, contributing to a growing but still limited body of research on the safety of digital and wearable-based/digital interventions in healthcare settings.

Although this is a small-scale pilot, the insights gained from the study will lay the foundation for future trials involving larger and more diverse samples. If proven feasible and acceptable, *WeCare* has the potential to offer an effective, scalable, and sustainable approach to improving stress resilience and mental wellbeing in high-risk healthcare environments, ultimately contributing to better care, reduced costs, and a more resilient health workforce.

### Strengths and Limitations

The study is highly innovative and demonstrates several notable strengths. It targets under-served, high-risk populations with limited access to preventative mental health support. The intervention is novel in that it combines safe and widely accepted consumer-grade wearable technology with evidence-based stress management and wellbeing strategies delivered through digital tools, providing a scalable and low-cost model. A staggered multiple-baseline design and sequential mixed methods strengthened internal validity and allow for a nuanced understanding of user engagement and outcomes. Importantly, the study adopts a collaborative approach, incorporating input and feedback from end-users to co-design solutions that are acceptable, accessible, affordable, and beneficial, ensuring that the tools are tailored to real-world needs and preferences for sustained use [51]. As this paper describes a protocol rather than a completed study, these strengths relate to the proposed methodological approach and the anticipated value of the planned research. This protocol lays the foundation for a future fully powered trial and supports progression to subsequent implementation research.

While innovative, this study has limitations. Selection bias may occur due to the requirement for English proficiency, smartphone access, and digital literacy, potentially limiting participation from more diverse or disadvantaged groups. As expected in pilot research, where the main focus is on test feasibility, refining the intervention, and informing future trial design, the small planned sample size reflects the study’s exploratory nature and limits both generalisability and the ability to detect statistically significant effects. Adherence may also be influenced by the self-guided nature of the program, as digital interventions are often associated with higher attrition rates. To mitigate this, we incorporated both human and digital support strategies, including automated reminders, weekly check-ins, and engagement tracking. Potential expectation-related bias will also be addressed and minimised through transparent communication of the study’s exploratory aims during recruitment and onboarding.

## 4. Conclusions

Emergency care settings are inherently high-stress environments that present unique challenges for both patients and healthcare staff. By combining physiological monitoring with self-guided interventions delivered through personalised feedback, *WeCare* offers an innovative, wearable-integrated solution with the potential to enhance resilience, prevent serious mental health issues, and improve long-term wellbeing of vulnerable groups in EDs. This pilot study will generate critical evidence for wearable-supported wellbeing interventions, informing future research, service design, and clinical practice in emergency care settings.

## Figures and Tables

**Table 1:** Detail of experimental phases.

Phase	When It Starts	How Long	Device Used	What Happens
Baseline (Usual care)	*Sample* 1 and 3: 0–2 weeks post-ED care. *Sample* 2: 4–6 weeks post-ED care. *Sample* 4: Any time.	1–3 weeks (7, 11, 15 days by tier)	“Blinded” smartwatch (wrist device)	- In-person onboarding. - Wear smartwatch daily. - Smart ring sized for next phase. - Continue usual care, no changes. - Daily diaries and weekly surveys.
WeCare Program (Intervention)	After baseline phase ends	6 weeks (staggered by tier)	“Blinded” smartwatch Smart ring	- In-person onboarding. - Continue smartwatch monitoring. - Start intervention using a digital health platform and a smart ring as companions to track real-time physiological data and receive personalised education/feedback.
Optional Extension (Device testing)	After intervention phase ends	2 weeks	“Blinded” smartwatch	- Smartwatch (instead of smart ring) as intervention companion. - Test delivery method only. - Part of qualitative, co-design acceptability analysis.

**Table 2:** Participants’ inclusion and exclusion criteria.

Participant Group	Inclusion Criteria	Exclusion Criteria
All	Aged 18–70 years. Sufficient English proficiency. Willing to wear a smart watch and a smart ring * device for at least 22 hours per day for the next 3 months. * Comfort or infection-control issues related to wearing hand/wrist devices will be monitored and addressed as needed.	History or evidence of substance abuse (e.g., alcohol or illicit drugs) at the time of admission. History of severe mental health disorders, e.g., bipolar disorder or psychoses or chronic depression disorder, determined by medical history or interview. History of unstable or poorly controlled cardiovascular, neurological, or other serious systemic disease that is likely to interfere with study evaluation or participant safety (e.g., uncontrolled blood pressure, unstable arrhythmia, decompensated heart failure, recent myocardial infarction or stroke, and new or uncontrolled metabolic or inflammatory conditions). Evidence of any conditions (e.g., cognitive disorder or dementia) that interfere with the patient’s ability to understand the requirements of the study and their ability to consent. Undergoing, or planning to undergo, surgery or inpatient admission/hospitalisation within the next three months.
Mild-to-moderate injury patient group (*Sample* 1)	Sustained in last 2 weeks a minor-to-moderate injury due to traumatic unintentional cause (e.g., traffic crash, fall, or work-related), such as a whiplash injury, minor fractures, limb fractures, or joint sprains or strains. Presented to RNSH emergency department (ED), Sydney, Australia. Injury Severity Score (ISS) < 15.	Injuries due to assaults or self-harm, because they are often associated with severe additional psychological stress or trauma. Injuries involving the brain. Very minor, localised, superficial soft-tissue injuries, such as small cuts, abrasions, minor bruises, or localised swelling.
Severe injury patient group (*Sample* 2)	Sustained in last 4 weeks a severe traumatic injury due to non-violent cause (e.g., traffic crash, fall, or work-related), such as multi-trauma or severe injuries requiring aggressive therapy and/or admission to the Intensive Care Unit (ICU). Presented to RNSH ED, Sydney, Australia. ISS ≥ 15.	Injuries due to assaults or self-harm. Injuries involving the brain. Very minor, localised, superficial soft-tissue injuries, such as small cuts, abrasions, minor bruises, or localised swelling.
Medical patient group (*Sample* 3)	Acute onset of medical symptoms of minor-to-moderate severity, such as gastrointestinal symptoms (e.g., abdominal pain or nausea), urinary symptoms (e.g., urinary tract infections), respiratory symptoms (e.g., mild shortness of breath or persistent cough), migraine, or non-traumatic musculoskeletal symptoms (e.g., chronic or spontaneous back pain, neck pain), prompting an ED visit in last 2 weeks. Presented to RNSH ED, Sydney, Australia. Received a green triage code (Australasian Triage Scale (ATS) category 3–5 = less urgent). Requiring standard treatment home or inpatient non-ICU.	Received a yellow, red, or blue triage code, requiring aggressive or palliative treatment, intubation, or ICU admission. Trauma-related musculoskeletal conditions (e.g., fracture, whiplash, or sprain from a fall/crash).
ED healthcare staff (*Sample* 4)	Being a healthcare worker at the ED of RNSH, Sydney, Australia, including medical doctors, nurses, paramedics, and allied health professionals.	History of injury requiring ED presentation or exposure to a traumatic event (i.e., perceived threat of injury to self or others) in the past year. Presented to the ED or admitted to hospital in the past year.

ATS: Australasian Triage Scale; ED: Emergency Department; ICU: Intensive Care Unit; ISS: Injury Severity Score; RNSH: Royal North Shore Hospital.

**Table 3:** Description of study outcomes for the experimental component.

**(a) PRE-BASELINE MEASURES**	OUTCOME	Data Collection	Day 0	Daily	Ecological *	Weekly	Pre-Program	Post-Program	Follow-Ups
Socio-demographic	Seven multiple-choice questions	Online/Paper	✓	-	-	-	-	-	-
Clinical	Medical and mental health history	Online/Paper	✓	-	-	-	-	-	-
	Treatment and medication use	Online/Paper	✓	-	-	-	-	-	-
	Lifestyle habits	Online/Paper	✓	-	-	-	-	-	-
Psychosocial	PSI stress	Online/Paper	✓	-	-	-	-	-	-
	PSI wellbeing	Online/Paper	✓	-	-	-	-	-	-
	PSI psychological distress	Online/Paper	✓	-	-	-	-	-	-
	PSI abnormal illness behaviour	Online/Paper	✓	-	-	-	-	-	-
	PSI quality of life	Online/Paper	✓	-	-	-	-	-	-
	Pain Catastrophising Scale	Online/Paper	✓	-	-	-	-	-	-
	Injustice Experience Questionnaire	Online/Paper	✓	-	-	-	-	-	-
	Composite Scale of Morningness	Online/Paper	✓	-	-	-	-	-	-
	Brief Resilience Scale	Online/Paper	✓	-	-	-	-	-	-
	General Self-Efficacy Scale	Online/Paper	✓	-	-	-	-	-	-
**(b) PRIMARY OUTCOMES**	OUTCOME	Data collection	Day 0	Daily	Ecological *	Weekly	Pre-program	Post-program	Follow-ups
Mental health status	Subjective Units of Distress Scale	Online/Paper	✓	✓	✓	-	-	✓	✓
Sleep quality	Numeric Rating Scale (0 to 10)	Online/Paper	✓	✓	-	-	-	✓	✓
Autonomic function	HRV metrics	Wearable devices	-	✓ **	-	-	-	✓ **	✓ **
Sleep function	Sleep metrics	Wearable devices	-	✓ **	-	-	-	✓ **^	✓ **^
**(c) GENERALISATION MEASURES**	OUTCOME	Data collection	Day 0	Daily	Ecological *	Weekly	Pre-program	Post-program	Follow-ups
‘Proximal’ Generalisation Measures									
Mental health function	Perceived Stress Scale	Online/Paper	✓	-	-	✓	-	✓	✓
	Patient Health Questionnaire	Online/Paper	✓	-	-	✓	-	✓	✓
	PTSD Checklist for DSM-5	Online/Paper	✓	-	-	✓	-	✓	✓
	Generalised Anxiety Disorder Questionnaire	Online/Paper	✓	-	-	✓	-	✓	✓
Sleep	The Pittsburgh Sleep Quality Index	Online/Paper	✓	-	-	✓	-	✓	✓
Adverse events and life stressors	Self-reported adverse events and stressors diaries	Online/Paper	✓	-	-	✓	-	✓	✓
Consumer perception	Numeric Rating Scale (−7 to +7)	Online/Paper	✓	-	-	✓	-	✓	✓
Quality of life	EQ-5D-5L—Health score (0 to 100)	Online/Paper	✓	-	-	✓	-	✓	✓
Physiological status	HR, RR, Sp02, activity, summary scores, temp	Wearable devices	-	✓ **	-	-	-	✓ **^	✓ **^
‘Distal’ Generalisation Measures									
Mental wellbeing	The World Health Organisation-Five Well-Being Index	Online/Paper	-	-	-	-	✓	✓	✓
Cognitive functioning	Perceived Deficits Questionnaire	Online/Paper	-	-	-	-	✓	✓	✓
Pain interference	Numeric Rating Scales (0 to 10)	Online/Paper	-	-	-	-	✓	✓	✓
Fatigue	Numeric Rating Scales (0 to 10)	Online/Paper	-	-	-	-	✓	✓	✓
Social participation	WHODAS 2.0 Participation Domain	Online/Paper	-	-	-	-	✓	✓	✓
Consumer satisfaction	Was-It-Worth-It Questionnaire	Online/Paper	-	-	-	-	✓	✓	✓
Continuation of use	Three custom questions	Online/Paper	-	-	-	-	-	✓	✓

* Every 2 h for a maximum of 4 days (2 during baseline phase, 2 during intervention phase); ** Continuous monitoring using wearables; ^ If participants are willing to continue the wearable-based self-monitoring past the 11 weeks. ✓ Measured at the specific time point.

## Data Availability

Data supporting the findings of this study will be made available from the authors upon reasonable request.

## Abbreviations

The following abbreviations are used in this manuscript:

ANS	Autonomic nervous system
ATS	Australasian Triage Scale
ED	Emergency Department
GAD-7	Generalised Anxiety Disorder Scale
HRV	Heart rate variability
ICU	Intensive Care Unit
ISS	Injury Severity Score
PCL-5	PTSD Checklist for DSM-5
PHQ-9	Patient Health Questionnaire-9
PSS	Perceived Stress Scale
PTSD	Post-traumatic stress disorder
PSI	Psychosocial Index
PSQI	Pittsburgh Sleep Quality Index
rMSSD	Root Mean Square of Successive Differences
RNSH	Royal North Shore Hospital
RoBiNT	Risk of Bias in N-of-1 Trials
SUDS	Subjective Units of Distress Scale
TFA	Theoretical Framework of Acceptability
